# Neural pathways of phonological and semantic processing and its relations to children’s reading skills

**DOI:** 10.3389/fnins.2022.984328

**Published:** 2022-10-12

**Authors:** Neelima Wagley, James R. Booth

**Affiliations:** Department of Psychology and Human Development, Vanderbilt University, Nashville, TN, United States

**Keywords:** phonology, semantics, word reading, reading comprehension, fMRI

## Abstract

Behavioral research shows that children’s phonological ability is strongly associated with better word reading skills, whereas semantic knowledge is strongly related to better reading comprehension. However, most neuroscience research has investigated how brain activation during phonological and semantic processing is related to word reading skill. This study examines if connectivity during phonological processing in the dorsal inferior frontal gyrus (dIFG) to posterior superior temporal gyrus (pSTG) pathway is related to word reading skill, whereas connectivity during semantic processing in the ventral inferior frontal gyrus (vIFG) to posterior middle temporal gyrus (pMTG) pathway is related to reading comprehension skill. We used behavioral and functional magnetic resonance imaging (fMRI) data from a publicly accessible dataset on OpenNeuro.org. The research hypotheses and analytical plan were pre-registered on the Open Science Framework. Forty-six children ages 8–15 years old were included in the final analyses. Participants completed an in-scanner reading task tapping into phonology (i.e., word rhyming) and semantics (i.e., word meaning) as well as standardized measures of word reading and reading comprehension skill. In a series of registered and exploratory analyses, we correlated connectivity coefficients from generalized psychophysiological interactions (gPPI) with behavioral measures and used *z*-scores to test the equality of two correlation coefficients. Results from the preregistered and exploratory analyses indicated weak evidence that functional connectivity of dIFG to pSTG during phonological processing is positively correlated with better word reading skill, but no evidence that connectivity in the vIFG-pMTG pathway during semantic processing is related to better reading comprehension skill. Moreover, there was no evidence to support the differentiation between the dorsal pathway’s relation to word reading and the ventral pathway’s relation to reading comprehension skills. Our finding suggesting the importance of phonological processing to word reading is in line with prior behavioral and neurodevelopmental models.

## Introduction

Reading is facilitated by three main interconnected systems: orthography, phonology, and semantics involving the occipitotemporal, temporoparietal, and inferior frontal cortex (Harm and Seidenberg, 2004; Sandak et al., 2012). Prior work, predominantly based on languages with alphabetic scripts, has established that the functional architecture of this network is associated with different reading skills throughout development (see Pugh et al., 2010; Landi et al., 2013 for review). Relations between network engagement and reading ability are commonly used to characterize differences in individuals with reading difficulties (e.g., Hoeft et al., 2006; van der Mark et al., 2011; Norton et al., 2015) and in skilled readers (e.g., Turkeltaub et al., 2003; Jobard et al., 2011; Welcome and Joanisse, 2012; Aboud et al., 2016; Ryherd et al., 2018). However, most neurobiological theories and extant computational models examine reading outcomes at the single-word level (Seidenberg, 2012; Pugh et al., 2013). In the current study, we examine how engagement of the neural pathways for phonological and semantic processing are related to individual differences in word reading versus reading comprehension skills in children ages 8–15 years old.

In alphabetic languages, successful word reading skills are strongly associated with phonological awareness abilities whereas reading comprehension skills are strongly associated with semantic knowledge (e.g., Wagner and Torgesen, 1987; Melby-Lervåg et al., 2012; Lervåg et al., 2018; Hjetland et al., 2019). Phonological skills are particularly relevant during the early stages of reading acquisition when children heavily rely on phoneme awareness and letter knowledge to decode words (Hjetland et al., 2019). They may also be involved in learning to read in non-alphabetic orthographies such as Chinese (e.g., McBride-Chang and Suk-Han Ho, 2005). However, this study is restricted to considering the role of phonological skills in learning to read in English. Patterns of neurodevelopment also suggest that successful reading is initially supported by brain connectivity for phonological decoding with a decreased reliance on this strategy as reading becomes more automated (Harm and Seidenberg, 2004; Shaywitz et al., 2004; Martin et al., 2015; Younger et al., 2017). Together, word decoding and language comprehension skills accounts for a large percent of variance in concurrent reading comprehension skills and its growth over time (Gough and Tunmer, 1986; Lervåg et al., 2018; Hjetland et al., 2019). Individuals with reading comprehension deficits, despite adequate phonemic decoding skill, can have difficulty with word-level semantic processing (e.g., Nation and Snowling, 1999; Landi and Perfetti, 2007; Cutting et al., 2013; Henderson et al., 2013; Spencer et al., 2014) and with higher-level word to text integration (Oakhill and Cain, 2012; Silva and Cain, 2015). Thus, assessing how the neural pathways for phonological and semantic processing relate to reading beyond single words may inform targeted remediation strategies and contribute to understanding long-term literacy outcomes (Landi and Ryherd, 2017).

Multiple overlapping and distinct brain regions support phonological and semantic processing (e.g., Fiebach et al., 2002; Vigneau et al., 2006; Mathur et al., 2020; Hodgson et al., 2021). Research suggests that the fronto-temporal network for reading is evident in children as young as 4 years old (Mathur et al., 2020; Jasińska et al., 2021). In a recent meta-analysis by Hodgson et al. (2021) comparing the two, phonological processing primarily involved a large cluster in the frontal lobe including the precentral gyrus and inferior frontal gyrus (IFG) pars opercularis and left posterior superior temporal gyrus (STG), as well as the superior parietal lobe. These hubs make-up the *dorsal* pathway of the reading circuitry. By contrast, the *ventral* pathway is associated with semantic processing and involves clusters in the left IFG pars triangularis and orbitalis and left posterior middle temporal gyrus (MTG), as well as the left anterior temporal lobe and angular gyrus (Hodgson et al., 2021). Prior cross-modal work suggests that these regions are generally engaged during phonological and semantic processing irrespective of the visual or auditory modality (e.g., Booth et al., 2002; Landi et al., 2010; Williams et al., 2015; Oron et al., 2016).

Prior work suggests a functional separation of the dorsal versus ventral left IFG for phonological and semantic processing (e.g., Jobard et al., 2003; Coltheart, 2005; Vigneau et al., 2006; Mathur et al., 2020; Hodgson et al., 2021; Wang et al., 2021b). Phonological processing of speech sounds involves perceptual processing in the STG and articulatory processing in the dorsal IFG (Hickok and Poeppel, 2004, 2007). Specifically, the dorsal IFG accesses phonological representations through connections via the arcuate fasciculus with STG (Saur et al., 2010; Boets et al., 2013) and is specialized for phonological processing during language production (Vigneau et al., 2006; Klaus and Hartwigsen, 2019). Specialization of the dorsal IFG for phonological processing is also evident when using a visual word rhyming task (e.g., Mathur et al., 2020). The MTG is engaged in lexical-semantic processes while the ventral IFG supports controlled processes such as meaning judgments or plausibility categorization (Thompson-Schill et al., 1997, 1999; Badre et al., 2005; Binder et al., 2009; Friederici and Gierhan, 2013). The ventral IFG accesses stored semantic knowledge in the temporal cortex through connections via the uncinate fasciculus (Thompson-Schill et al., 1997; Lau et al., 2008). It appears that these interconnected regions involved in phonological and semantic processing become more specialized with increased language experience (e.g., Weiss-Croft and Baldeweg, 2015; Skeide and Friederici, 2016; Perrone-Bertolotti et al., 2017).

Across studies using auditory and visual rhyming paradigms, phonological specialization in the left STG is evident in children by age five (e.g., Weiss et al., 2018; Mathur et al., 2020; Yamasaki et al., 2021), whereas specialization in the dorsal IFG is thought to develop later around age seven (e.g., Wang et al., 2021b; Yamasaki et al., 2021). Notably, the engagement of this *dorsal* fronto-temporal pathway during phonological processing is predictive of children’s word reading skills throughout reading development (Wang et al., 2013). During an auditory word rhyming task, there was significant activation in the posterior left STG in children 6-years-old (Wang et al., 2020) and in the left IFG pars opercularis and posterior STG in children 7.5-years-old (Wang et al., 2021a). In the younger children, phonological processing in the left STG was a significant predictor of word reading skills 1.5 years later, even after controlling for initial levels of reading (Wang et al., 2020). In the older children, stronger functional connectivity from the dorsal IFG to STG during phonological processing predicted better word reading skills 1.5 years later (Wang et al., 2021a). These findings suggest that, by early elementary school, dorsal IFG and STG are specialized for phonological processing and that effective access of phonological representations via this pathway scaffolds children’s word reading development (Wang et al., 2021a).

Semantic specialization in the left MTG is also evident in children by age five (e.g., Mathur et al., 2020; Wang et al., 2021b), whereas specialization in the ventral IFG seems to develop later around age seven (e.g., Wang et al., 2021b). Across studies with skilled adult and younger readers, engagement of the *ventral* fronto-temporal pathway during semantic processing is related to discourse-level reading skills (e.g., Lee et al., 2016; Yu et al., 2018; Jasińska et al., 2021). During a word reading task in adults, reading comprehension skill was significantly related to brain activation in the left IFG pars triangularis (Malins et al., 2016) and in the left MTG (Welcome and Joanisse, 2012). In adolescents ages 12–18 years, skilled comprehenders showed greater activation in left IFG pars triangularis and bilateral MTG during a discourse comprehension task (e.g., Landi et al., 2010; Ryherd et al., 2018). Together, this literature suggests that the ventral IFG and MTG are reliably engaged during both word- and discourse-level semantic tasks, and those with poor comprehension skills often struggle with accessing the lexical-semantic representations via this pathway during reading (e.g., Cutting et al., 2013).

In a recent study using a word rhyming and a word meaning task, better readers showed greater engagement of the dorsal IFG (pars opercularis) during phonological processing (*r* = 0.40) and a trend for greater engagement of the ventral IFG (pars triangularis) during semantic processing (*r* = 0.30; Brozdowski and Booth, 2021, *preprint*). However, reading skill was only assessed at the single-word level. The current study builds on the prior literature suggesting that phonological ability is strongly associated with better word reading skills, whereas semantic knowledge is strongly related to better reading comprehension skills. This study is the first to directly compare brain-behavior correlations to test how the engagement of the dorsal and ventral pathways may differentially relate to word- and passage- level reading skills. Specifically, we examine if the engagement of the dorsal pathway (i.e., dIFG to pSTG) during phonological processing is related to word reading skill, whereas the engagement of the ventral pathway (i.e., vIFG to pMTG) during semantic processing is related to reading comprehension skill in children ages 8–15 years old.

Based on the prior literature, we hypothesized the following: (1) the correlation between connectivity in the dorsal pathway (dIFG-pSTG) and word reading skills will be stronger than the correlation between connectivity in the ventral pathway (vIFG- pMTG) and word reading skills, (2) the correlation between connectivity in the ventral pathway (vIFG-pMTG) and reading comprehension skills will be stronger than the correlation between connectivity in the dorsal pathway (dIFG-pSTG) and reading comprehension skills, (3) the correlation between word reading skills and connectivity in the dorsal pathway (dIFG-pSTG) will be stronger than the correlation between reading comprehension skills and connectivity in the dorsal pathway (dIFG-pSTG), and (4) the correlation between reading comprehension skills and connectivity in the ventral pathway (vIFG-pMTG) will be stronger than the correlation between word reading skills and connectivity in the ventral pathway (vIFG-pMTG).

## Materials and methods

This study was conducted used the Cross-Sectional Multidomain Lexical Processing dataset available on (Lytle et al., 2020). The research questions, hypotheses, and analytical plan were preregistered through the Open Science Framework after data cleaning but prior to beginning the data analyses.^1^

### Participants

The dataset contains a sample of 91 native English-speaking children with normal hearing and normal or corrected-to-normal vision, with no neurological or psychiatric disorders, and not taking medications impacting the central nervous system. Data from participants who met the following inclusionary criteria were analyzed for the current study: (1) primarily right-handed assessed using five actions of writing, drawing, picking-up, opening, and throwing; score ≥ 3 indicates right-handedness (N = 4 excluded); (2) a standard score of at least 70 on the performance IQ subscale of the Wechsler Abbreviated Scale of Intelligence (WASI-II; Wechsler, 1999; N = 2 excluded); and (3) having complete behavioral and imaging data with limited movement and acceptable task performance in the scanner (N = 38 excluded, see details below). One additional participant was excluded for errors found during data pre-processing. Forty-six participants are included in the final analyses (M_*age*_ = 11.7, SD = 2.2, 25 females, see Table 1). A list of included participant IDs is reported in the Supplementary Table 1. The excluded participants had comparable non-verbal IQ (*t* = 0.92, *p* = 0.32), word reading (*t* = 1.23, *p* = 0.32), and reading comprehension (*t* = 1.52, *p* = 0.13) scores to participants that were included in the final analysis.

**TABLE 1:** Participant demographics, standard scores on reading assessments, and behavioral performance during the two functional magnetic resonance imaging (fMRI) tasks.

	N = 46 (25 females)			
	M (SD)		Range	
Age in years	11.7 (2.2)		8.7–15.5	
WASI-II non-verbal IQ	106 (15)		77–144	
WJ-III letter word ID	105 (12)		82–130	
WJ-III passage comp.	103 (11)		76–133	

	**Accuracy (%)**		**Response time (ms)**	
	**M (SD)**	**Range**	**M (SD)**	**Range**

*Rhyming task*				
O+P+	90 (11)	58–100	1,391 (347)	716–2,177
O–P+	78 (19)	21–100	1,462 (339)	815–2,392
O+P–	64 (25)	4–100	1,563 (359)	935–2,398
O–P–	93 (11)	44–100	1,390 (375)	705–2,293
Fixation	96 (7)	68–100	1,354 (357)	677–2,044
*Meaning task*				
Strongly related	92 (12)	54–100	1,344 (338)	588–2,158
Weakly related	89 (13)	46–100	1,331 (317)	656–2,122
Unrelated	90 (14)	42–100	1,466 (375)	764–2,337
Fixation	97 (7)	62–100	1,367 (326)	680–1,980

Participants were recruited from the greater Chicago area. In total, 13% of the participants identified as Hispanic or Latinx. In total, 67% of participants identified as White, 11% as Black or African American, 9% as “other”, 9% as multiracial, and 4% as Asian. Caregivers and children completed informed consent and assent forms before participation. All study procedures were approved by the Institutional Review Board at Northwestern University and Evanston Northwestern Healthcare Research Institute.

### Procedure

Participants completed behavioral and fMRI tasks over two or more visits. First, participants completed standardized behavioral assessments followed by a practice imaging session in a mock scanner within a week of their fMRI session. This allowed participants to become familiar with the in-scanner tasks as well as the scanning environment. Practice tasks did not include any stimuli used in the experimental tasks. Lastly, participants completed the fMRI sessions.

### Behavioral assessments of reading

We used raw scores from the *Word Identification* and *Passage Comprehension* subtests of the Woodcock-Johnson III Tests of Achievement (WJ-III; Woodcock et al., 2001) to assess word reading and reading comprehension skills, respectively. Six participants from the original dataset were excluded for not having complete reading data. The *Word Identification* subtest involves reading a series of words aloud, arranged from low- to high- difficulty, with a total of 76 items. Standard scores on the word reading task for the selected participants ranged from 82 to 130 [M(SD) = 105 (11)]. The Passage Comprehension subtest involves reading short sentences and identifying a missing key word that made sense in the context of the passage, with a total of 47 items. Standard scores on the reading comprehension task for the selected participants ranged from 76 to 133 [M(SD) = 103 (11)].

### Functional magnetic resonance imaging lexical judgment tasks

Participants completed a rhyming task and a meaning task in the scanner. For both tasks, two words were visually presented in sequential order and contained three condition types: lexical, perceptual control, and fixation.

In all trials, the first stimulus was presented for 800 ms followed by an intertrial interval of 200 ms and the second stimulus for 800 ms. The second stimulus was followed by a red fixation cross lasting 2,600 ms indicating that participants should respond. Participants could respond as soon as the second stimulus was presented up until the start of the next trial. The second stimulus was offset right or left 1/2 a letter/symbol from the first to prevent judgments based on visual persistence. Stimuli were presented in the same order for all participants, optimized for event-related design using OptSeq (Greve, 2002). Word characteristics are provided in the stimuli directory of the OpenNeuro dataset (Lytle et al., 2020).

In the *Rhyming Task*, participants read two words and judged if the pair of words rhymed. Word pairs were grouped into four lexical conditions with 24 pairs in each condition: orthographically similar and phonologically similar (O+P+, *gate-hate*), orthographically different and phonologically similar (O–P+, *has-jazz*), orthographically similar and phonologically different (O+P–, *pint-mint*), and orthographically different and phonologically different (O–P–, *press-list*). Trial order was optimized and divided into two 108 trial runs collected in 240 volumes. All but five participants completed both runs in the same scanning session.

In the *Meaning Task*, participants read two words and judged if the pair of words were related in meaning. Word pairs were grouped into three conditions based on free association values (Nelson et al., 1998) with 24 pairs in each condition: strongly related (*found-lost*), weakly related (*dish-plate*), and unrelated (*tank-snap*). The average strength of association between word pairs in the strongly related condition was 0.60 (range = 0.36–0.77), 0.30 in the weakly related condition (range = 0.14–0.60), and 0 in the unrelated condition. Six word pairs from the weakly related condition overlapped in association values (>0.36) with the strongly related condition. Trial order was optimized and divided into one run with 91 trials and a second run with 89 trials. Due to the difference in length, run 1 for was collected in 203 volumes and run 2 was collected in 198 volumes. All but three participants completed both runs on the same scanning session.

In addition to the lexical trials, both tasks contained perceptual control and fixation trials. Participants were presented with a pair of symbols and were asked if the pair matched or not. Perceptual control trials were not modeled as conditions of interest in the present study. The fixation condition controlled for motor responses. In these trials, participants were presented with a black fixation during the first and second stimulus phases and a red fixation during the response phase. Participants were asked to press the button under their index finger when the black cross turned red. The number of trials for the perceptual and fixation trials were as follows: 24 matching perceptual trials, 24 non-matching perceptual trials, and 72 fixation control trials.

Only those with complete data for both runs of the two tasks were included in the analysis (N = 20 excluded for missingness). Additionally, those who scored within an acceptable accuracy range and had no response bias were included in the analysis. Acceptable accuracy was defined as at least 50% accuracy in the O+P+, strongly related, and fixation conditions (N = 5 excluded). The lack of response bias was defined by no greater than a 50% difference in accuracy between the O+P+ and O–P– conditions for the rhyming task and the strongly related and unrelated conditions for the semantic task (N = 1 excluded).

### Functional magnetic resonance imaging data acquisition

Magnetic resonance data were acquired using a 1.5 T General Electric (GE) Signa Excite scanner at Evanston Hospital, using a quadrature birdcage head coil. Participants were placed supine in the scanner and their head position was secured using a vacuum pillow (Bionix, Toledo, OH, USA). A response box was placed in the participant’s right hand to allow them to respond to the tasks. Task stimuli were projected onto a screen, which the participants viewed through a mirror attached to the inside of the head coil. Structural T1-weighted SPGR images were collected using the following parameters: TR 1/4 33.333 ms, TE 1/4 8 ms, matrix size 1/4 256 × 256, bandwidth 1/4 114.922 Hz/Px, slice thickness 1/4 1.2 mm, number of slices 1/4 124, voxel size 1/4 1 mm isotropic, flip angle 1/4 30°. Blood oxygen level dependent signal (BOLD) was acquired using a T2-weighted susceptibility weighted single-shot echo planar imaging (EPI) and the following parameters: TR 1/4 2,000 ms, TE 1/4 25 ms, matrix size 1/4 64 × 64, bandwidth 1/4 7812.5 Hz/Px, slice thickness 1/4 5 mm, number of slices 1/4 24, voxel size 1/4 3.75 mm × 3.75 mm × 5 mm, flip angle 1/4 90°. Slices were acquired interleaved from bottom to top, odd first.

### Functional magnetic resonance imaging data analysis

#### Preprocessing

Functional magnetic resonance imaging data was analyzed using SPM12.^2^ Images were spatially realigned to the mean functional volume to correct for head movements, co-registered to the corresponding skull stripped T1 anatomical image and normalized to the Montreal Neurological Institute (MNI) space standard, with voxel size 2 mm^3^ × 2 mm^3^ × 2 mm^3^. Functional images were then spatially smoothed with a 6-mm full-width at half-maximum isotropic Gaussian kernel. We used ArtRepair (Mazaika et al., 2007) to detect outlier volumes with more than 1.5 mm volume-to-volume movement, or with more than 4% deviation from the mean global signal. Outlier volumes were repaired by interpolating between the nearest non-outlier volumes. Interpolated volumes were then de-weighted when calculating first-level models on repaired images (Mazaika et al., 2007). No more than 10% of the volumes from each run and no more than six consecutive volumes for any individual were interpolated in this way. Six participants were excluded from analysis for excessive movement.

#### First-level analysis

First-level statistical analyses were performed on individual participants’ data using the general linear model (GLM) as implemented in SPM12. The following regressors were entered into the GLM for the two runs: six motion regressors of head movement, two perceptual control conditions of no interest, one fixation condition, and four rhyme (O+P+, O+P–, O–P+, and O–P–) and three meaning (strongly related, weakly related, and unrelated) lexical conditions, for each run. The contrast of lexical > fixation was defined to produce individual level activation maps, which include the four rhyme or three meaning conditions.

#### Regions of interest masks

Based on the prior literature, four anatomical masks were used to isolate the ROIs for each task: left dorsal inferior frontal gyrus (dIFG; pars opercularis) and left posterior superior temporal gyrus (pSTG) for phonology and the left ventral inferior frontal gyrus (vIFG; pars triangularis and pars orbitalis) and left posterior middle temporal gyrus (pMTG) for semantics (Hodgson et al., 2021). The regions were identified using the anatomical automatic labeling (AAL) atlas template from WFU PickAtlas toolbox^3^ and the MarsBar toolbox (Brett et al., 2002). The pSTG was defined as the posterior half of the left STG with *y* < −24 and the pMTG was defined as the posterior half of the left MTG with *y* = −33.

#### General psychophysiological interaction analysis

For each task, the top 100 voxels showing maximal activation (regardless of significance) for each participant for the lexical > fixation contrast in the dIFG (for the rhyming task) and vIFG (for the meaning task) was used as the seed region. We chose the top 100 voxels at the subject-level to define the seed region to focus on individual differences rather than a group-based cluster. This approach of using individualized ROIs is thought to be more sensitive at capturing the experimental manipulation and detecting differences between conditions and groups (Fedorenko et al., 2010; Tong et al., 2016). Specifically, the method of using the top 100 voxels regardless of significance, has been used by several previous studies to examine brain-behavioral correlations (Suárez-Pellicioni and Booth, 2018; Suárez-Pellicioni et al., 2019; Younger et al., 2019; Wang et al., 2020), including capturing individual differences in phonological and semantic processing using comparable paradigms of language and reading (e.g., Wang et al., 2021a; Yamasaki et al., 2021; Wang, 2022). Next, the following regressors were entered into a GLM in the individual level analysis for the two runs: the timeseries from the seed region, the experimental parameter regressors (seven for the rhyming task and six for the meaning task), the PPI regressors of the interaction (seven for the rhyming task and six for the meaning task), and the six motion regressors of head movement, for each run. The contrast of lexical > fixation was defined to produce individual level functional connectivity maps. Following, the average gPPI beta values were extracted from the top 100 voxels with the strongest connectivity in the pSTG (for rhyming) and pMTG (for meaning) anatomical mask. The dIFG-pSTG and vIFG-pMTG path coefficients for each participant were entered into the correlation analyses with reading scores. See Supplementary material for activation maps showing overlap across participants for the seed and target regions (Supplementary Figure 1) and the whole brain activation maps for the pre-registered (Supplementary Figure 2) and exploratory (Supplementary Figures 3–5) contrasts.

#### Brain and behavior analysis

Beta values from general psychophysiological interaction analysis (gPPI and raw scores from the reading assessments were entered into a partial correlation analysis using Pearson’s *r* with age a covariate (ppcor package in R; Kim, 2015). Each brain-behavior partial correlation was independently calculated prior to computing the comparisons of correlation coefficients. To test each hypothesis, we used an interactive calculator to compute the *z*-score between two correlation coefficients (Lee and Preacher, 2013).^4^ This calculator tests for the difference between two correlation coefficients obtained from the same dataset with the two correlations sharing one variable in common. Each test of equality between correlation coefficients was evaluated using a 1-tailed *p* < 0.05 threshold, given that we expected the correlations to be in a specific direction. For example, we expected that the correlation between connectivity in the dorsal pathway and word reading skills would be stronger, in the positive direction, than the correlation between connectivity in the ventral pathway and word reading skills.

## Results

### Preregistered analyses

**FIGURE 1:**
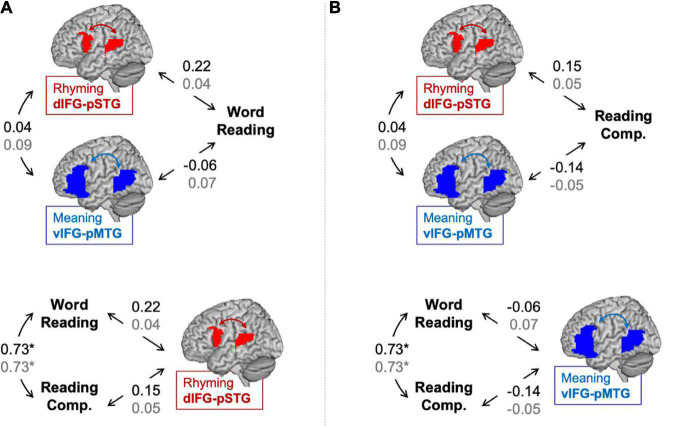
Partial correlations (controlling for age) between connectivity and *reading assessments* in the lexical > fixation (black) and the target-conditions > fixation (gray) contrasts. Panel **(A)** tests the hypotheses that the dorsal pathway for phonology is associated with word reading skill more than the ventral pathway for semantics and that word reading skill is associated with the dorsal pathway for phonology more than reading comprehension skill. Panel **(B)** tests the hypotheses that the ventral pathway for semantics is associated with reading comprehension skill more than the dorsal pathway for phonology and that reading comprehension skill is associated with the ventral pathway for semantics more than word reading skill. Statistically significant correlation denoted by an asterisk. *p < 0.001.

Partial correlations between connectivity during the lexical > fixation contrasts and reading skills are shown in Figure 1. No correlation is defined as *r* < 0.2 and weak correlation is defined as 0.2 < *r* < 0.4 (Dancey and Reidy, 2017). There was a weak correlation between word reading skill and dIFG-pSTG connectivity during the rhyming task (*r* = 0.22, *p* = 0.14), but no correlation between word reading skill and vIFG-pMTG connectivity during the meaning task (*r* = −0.06, *p* = 0.68). There was also no correlation between reading comprehension skill and dIFG-pSTG connectivity (*r* = 0.15, *p* = 0.34) or between reading comprehension skill and vIFG-pMTG connectivity (*r* = −0.14, *p* = 0.36); however, none of these correlations were significant. There was a strong correlation between the two reading skill measures (*r* = 0.73, *p* < 0.001), but no correlation between connectivity in the two pathways (*r* = 0.04, *p* = 0.81).

Results comparing correlation coefficients for each hypothesis are reported in Table 2 with weak evidence is defined as *z*-score > 1.0. There was weak evidence to support the hypotheses that word reading skill was associated with the dorsal pathway for phonological processing more than the ventral pathway for semantic processing (*z*-score = 1.36, *p* = 0.09). Contrary to our hypothesis, there was weak evidence to show that reading comprehension was associated with the dorsal pathway for phonological processing more than the ventral pathway for semantic processing (*z*-score = −1.37, *p* = 0.08). There was no evidence to support the hypotheses that the dorsal pathway was associated with word reading more than reading comprehension skill (*z*-score = 0.69, *p* = 0.24) and that the ventral pathway was associated with reading comprehension more than word reading skill (*z*-score = 0.72, *p* = 0.24).

**TABLE 2:** Results comparing correlation coefficients from the preregistered analysis (pre reg) using the lexical > fixation contrast (lex > fix) and exploratory analyses (explor 1 and explor 2) using the target-conditions > fixation (target > fix) contrasts.

Analysis	Contrast	Variable j	Variables k, h	*r*_jk_	*r*_jh_	*r*_kh_	*z*-score	1-tail *p*
pre reg	lex > fix	Word reading	dIFG-pSTG vIFG-pMTG	0.22	–0.06	0.04	1.36	0.09
pre reg	lex > fix	Reading comp.	dIFG-pSTG vIFG-pMTG	0.15	–0.14	0.04	–1.37	0.08
pre reg	lex > fix	dIFG-pSTG	Word reading Reading comp.	0.22	0.15	0.73	0.69	0.24
pre reg	lex > fix	vIFG-pMTG	Word reading Reading comp.	–0.06	–0.14	0.73	0.72	0.24
explor 1	target > fix	Word reading	dIFG-pSTG vIFG-pMTG	0.04	0.07	0.09	–0.15	0.44
explor 1	target > fix	Reading comp.	dIFG-pSTG vIFG-pMTG	0.05	–0.05	0.09	0.49	0.31
explor 1	target > fix	dIFG-pSTG	Word reading Reading comp.	0.04	0.05	0.73	–0.09	0.46
explor 1	target > fix	vIFG-pMTG	Word reading Reading comp.	0.07	–0.05	0.73	1.07	0.14
explor 2	lex > fix	Rhyme accuracy	dIFG-pSTG vIFG-pMTG	0.19	0.01	0.18	0.93	0.17
explor 2	lex > fix	Mean accuracy	dIFG-pSTG vIFG-pMTG	0.04	0.04	0.18	0.00	0.50
explor 2	lex > fix	dIFG-pSTG	Rhyme accuracy Mean accuracy	0.19	0.04	0.71	1.31	0.09
explor 2	lex > fix	vIFG-pMTG	Rhyme accuracy Mean accuracy	0.01	0.04	0.71	–0.26	0.40
explor 2	target > fix	Rhyme accuracy	dIFG-pSTG vIFG-pMTG	0.16	0.11	0.18	0.26	0.40
explor 2	targe > fix	Mean accuracy	dIFG-pSTG vIFG-pMTG	0.03	0.03	0.18	0.00	0.50
explor 2	target > fix	dIFG-pSTG	Rhyme accuracy Mean accuracy	0.16	0.03	0.71	1.13	0.13
explor 2	target > fix	vIFG-pMTG	Rhyme accuracy Mean accuracy	0.11	0.03	0.71	0.69	0.24

### Exploratory analyses 1 – Change in measurement of brain activation

In the first exploratory analysis, we changed the contrast of interest to increase specificity in the measurement of phonological and semantic processing in the brain. The rhyming task contains four lexical conditions of which two require a “yes” response (O+P+, O–P+) and two require a “no” response (O+P–, O–P–). The semantic task has three lexical conditions with two “yes” response conditions (strongly and weakly related) and one “no” response condition (unrelated). This mismatch in response types across the tasks may place different demands on language and cognitive processes and could lead to variations in brain activations and the localization of these effects. Additionally, participants’ task accuracy across the lexical conditions was higher for the semantic (average ∼90%) than the rhyming task (average ∼81%). To better equate the two experimental tasks across response type and difficulty, comparable task conditions were chosen for the exploratory analyses – O+P+ and O–P– for rhyming and strongly related and unrelated for meaning. The subsequent analyses using these conditions of interest will be referred to as the “target-conditions,” which also align with the task conditions used as part of the study’s inclusionary criteria for filtering accuracy and response bias.

In the first exploratory analysis, all first-level analysis parameters remained the same except the contrast of lexical > fixation was changed to [(O+P+ and O–P–) > fixation] for the rhyming task and [(strongly related and unrelated) > fixation] for the semantic task to produce individual level activation maps. The top 100 activated voxels (regardless of significance) in the dIFG and vIFG for these contrasts made up the seed regions for each task. Like the pre-registered analysis, we computed gPPI analysis using the timeseries from the newly defined contrasts to produce individual level functional connectivity maps. We extracted the average gPPI beta values from the top 100 voxels with the strongest connectivity in the pSTG and pMTG anatomical mask and computed brain-behavior analyses.

Partial correlations between connectivity during the target-conditions > fixation contrasts and reading assessments are shown in Figure 1. There was no correlation between word reading skill and dIFG-pSTG connectivity during the rhyming task (*r* = 0.04, *p* = 0.79) or between word reading skill and vIFG-pMTG connectivity during the meaning task (*r* = 0.07, *p* = 0.64). Similarly, there was no correlation of reading comprehension skill with dIFG-pSTG connectivity (*r* = 0.05, *p* = 0.72) or vIFG-pMTG connectivity (*r* = −0.05, *p* = 0.77). There was no correlation between connectivity in the two pathways using the new contrasts of interest (*r* = 0.09, *p* = 0.56).

Results comparing correlation coefficients for this exploratory analysis are reported in Table 2. There was no evidence to support the hypotheses that word reading skill was associated with the dorsal pathway for phonological processing more than the ventral pathway for semantic processing (*z*-score = −0.15, *p* = 0.44) or that reading comprehension skill was associated with the ventral pathway more than the dorsal pathway (*z*-score = 0.49, *p* = 0.31). There was no evidence to support the hypotheses that the dorsal pathway was associated with word reading more than reading comprehension skill (*z*-score = −0.09, *p* = 0.46). Contrary to our hypothesis, there was weak evidence to show that the ventral pathway for semantics was associated with word reading skill more than reading comprehension skill (*z*-score = 1.07, *p* = 0.14). However, this comparison was not statistically significant.

### Exploratory analyses 2 – Change in behavioral measure of reading skills

In the second exploratory analysis, we changed the behavioral assessments to increase specificity in the measurement of phonological and semantic processing during reading. While reading comprehension does engage vocabulary and activation of semantic knowledge, this measure additionally taps into cognitive processes such as integrating syntax and semantics, making inferences, self-monitoring, etc. (Melby-Lervåg and Lervåg, 2014). To better capture phonological processing as a core component of word reading skill and semantic processing as a central index of comprehension, we replaced the word reading and passage comprehension scores with performance on the in-scanner rhyming and meaning tasks, respectively.

All first-level and gPPI analysis parameters remained the same. Brain-behavior analyses used overall task accuracy as the outcome measure of reading. We used the Spearman rho to evaluate brain-behavior correlations as task accuracy data is non-parametric. We conducted this exploratory analysis using data from both the lexical > fixation and the target-conditions > fixation contrasts to parallel the prior analyses.

**FIGURE 2:**
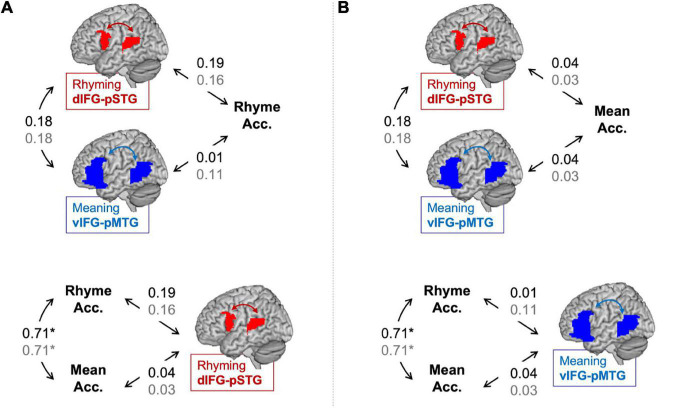
Partial correlations (controlling for age) between connectivity and *in-scanner task accuracy* in the lexical > fixation (black) and the target-conditions > fixation (gray) contrasts. Panel **(A)** tests the hypotheses that the dorsal pathway for phonology is associated with word rhyming accuracy more than the ventral pathway for semantics and that word rhyming accuracy is associated with the dorsal pathway for semantics more than word meaning accuracy. Panel **(B)** tests the hypotheses that the ventral pathway for semantics is associated with word meaning accuracy more than the dorsal pathway for phonology and that word meaning accuracy is associated with the ventral pathway for semantics more than word rhyming accuracy. Statistically significant correlation denoted by an asterisk. *p < 0.001.

Partial correlations between connectivity using the lexical > fixation contrasts and the in-scanner task accuracies are shown in Figure 2. There was no correlation between accuracy in the rhyming task and dIFG-pSTG connectivity during the rhyming task (*r* = 0.19, *p* = 0.22). There was no correlation between accuracy on the rhyming task and vIFG-pMTG connectivity during the meaning task (*r* = 0.01, *p* = 0.93). There was no correlation between accuracy on the meaning task and dIFG-pSTG connectivity (*r* = 0.04, *p* = 0.79) and vIFG-pMTG connectivity (*r* = 0.04 *p* = 0.82). There was a significant correlation between the two say in-scanner reading measures (*r* = 0.71, *p* < 0.001), and no correlation between connectivity in the two pathways (*r* = 0.18, *p* = 0.22). Like the preregistered analyses, none of these correlations were significant.

Partial correlations between connectivity using the target-conditions > fixation contrasts and the in-scanner task accuracies are shown in Figure 2. There was no correlation between accuracy in the rhyming task and dIFG-pSTG connectivity during the rhyming task (*r* = 0.16, *p* = 0.29) or vIFG-pMTG connectivity during the meaning task (*r* = 0.11, *p* = 0.46). There was no correlation between accuracy on the meaning task and dIFG-pSTG connectivity (*r* = 0.03, *p* = 0.84) or vIFG-pMTG connectivity (*r* = 0.03, *p* = 0.86).

Results comparing correlation coefficients for this exploratory analysis are reported in Table 2. Results were similar across the two analyses using the lexical > fixation and target-conditions > fixation contrasts. There was no evidence to support the hypotheses that accuracy on the word rhyming task was associated with the dorsal pathway for phonological processing more than the ventral pathway for semantic processing when using the lexical > fixation contrast (*z*-score = 0.93, *p* = 0.17) or the target-conditions > fixation contrast (*z*-score = 0.26, *p* = 0.40). There was no evidence that accuracy on the word meaning task was associated with the ventral pathway more than the dorsal pathway (*z*-score = 0, *p* = 0.50). There was weak evidence to support the hypotheses that the dorsal pathway for phonology was associated with accuracy on the word rhyming task more than accuracy on the word meaning task, for both the lexical > fixation (*z*-score = 1.31, *p* = 0.09) and target-conditions > fixation (*z*-score = 1.13, *p* = 0.13) contrasts. Lastly, there was no evidence that the ventral pathway for semantics was associated with accuracy on the word meaning task more than accuracy on the word rhyming task when using the target-conditions > fixation contrast (*z*-score = 0.69, *p* = 0.24) or the lexical > fixation contrast (*z*-score = −0.26, *p* = 0.40).

## Discussion

The primary focus of most cognitive neuroscience research in reading has been at the single-word-processing level. Deficits in phonological processing is a key marker of traditionally identified dyslexia, whereas deficits in semantic processing is thought to characterize specific deficits in reading comprehension (e.g., Rueckl and Seidenberg, 2009; Landi et al., 2010; Cutting et al., 2013). The aim of the current study was to examine if engagement of the dorsal pathway during phonological processing is related to word reading skill, whereas engagement of the ventral pathway during semantic processing is related to reading comprehension skill in children ages 8–15 years old. Results from the preregistered and exploratory analyses indicated weak evidence that dIFG to pSTG functional connectivity during phonological processing is positively correlated with word reading skill (see Table 3). The weak evidence is consistent with prior behavioral and neurodevelopmental models of reading suggesting that phonological awareness is associated with word reading ability (e.g., Melby-Lervåg et al., 2012; Pugh et al., 2013).

**TABLE 3:** Summary of results showing strength of evidence for each hypothesis across the pre-registered and exploratory analyses using the lexical > fixation (lex > fix) and target-conditions > fixation (target > fix) contrasts.

	Preregistered	Exploratory 1	Exploratory 2A	Exploratory 2B
	
	*lex* > *fix*	*target* > *fix*	*lex* > *fix*	*target* > *fix*
	*Reading skill*	*Reading skill*	*Task accuracy*	*Task accuracy*
(dIFG-pSTG ⇔ word reading/rhyming acc) > (vIFG-pMTG ⇔ word reading/rhyming acc)	Weak evidence	No evidence	No evidence	No evidence
(vIFG-pMTG ⇔ reading comp/meaning acc) > (dIFG- pSTG ⇔ reading comp/meaning acc)	Weak evidence for the alternate	No evidence	No evidence	No evidence
(word reading/rhyming acc ⇔ dIFG-pSTG) > (reading comp/meaning acc ⇔ dIFG-pSTG)	No evidence	No evidence	Weak evidence	Weak evidence
(reading comp/meaning acc ⇔ vIFG-pMTG) > (word reading/rhyming acc ⇔ vIFG-pMTG)	No evidence	Weak evidence for the alternate	No evidence	No evidence

Weak evidence is defined as *p* < 0.25.

The dorsal network’s engagement during phonological processing and its relation to word reading skills in English has been extensively examined across the range of reading development, from preliteracy to adolescence (e.g., Wang et al., 2013, 2020; Yu et al., 2018; Jasińska et al., 2021). A few studies have supported the argument that engagement of the ventral network for semantic processing is stronger in children with better reading comprehension skills (e.g., Landi et al., 2010; Cutting et al., 2013; Aboud et al., 2016; Ryherd et al., 2018). Overall, we observed that dorsal functional connectivity between dIFG and pSTG during phonological processing was weakly related to children’s word reading skill or accuracy on the visual rhyming task in the scanner. On the other hand, there was no evidence to support the hypothesis that the ventral pathway for semantics from vIFG to pMTG was related to reading comprehension skill or accuracy on the visual meaning task in the scanner. Lastly, there was no evidence to support the hypothesis that differential engagement of the dorsal and ventral pathways is related to word reading versus reading comprehension skill.

In the pre-registered analyses, we related functional connectivity during all lexical conditions with standardized measures of reading. Contrary to the prior literature showing moderate to strong associations between phonological brain systems and word reading skills (e.g., Wang et al., 2013, 2020; Yu et al., 2018; Jasińska et al., 2021; Yamasaki et al., 2021), we only found weak (and statistically unreliable) correlations between connectivity in the dorsal pathway and children’s word reading skills. To our surprise, this weak correlation was also evident in our first exploratory analyses which examined connectivity using a task contrast of targeted conditions, (O+P+ and O–P–) > fixation for the rhyming task and (strongly related and unrelated) > fixation for the semantic task. Some methodological parameters may help explain these limited findings.

We chose the IFG as a seed region because models of language and reading suggests a functional separation of the dorsal versus ventral left IFG for phonological and semantic processing, respectively (e.g., Mathur et al., 2020; Hodgson et al., 2021). Specifically, the dorsal IFG (pars opercularis) is thought to access the phonological representations in the STG through connections via the arcuate fasciculus, whereas the ventral IFG (pars triangularis and/or orbitalis) is believed to access stored semantic knowledge in the MTG through connections via the uncinate fasciculus (e.g., Badre et al., 2005; Saur et al., 2010; Friederici and Gierhan, 2013; Hodgson et al., 2021). In 7-year-old children, stronger functional connectivity from dorsal IFG to STG during phonological processing has been shown to predict better word reading skills later in development (Wang et al., 2021a). A recent study using the same word rhyming and meaning tasks as the current study found that better readers showed greater engagement of the dorsal IFG (pars opercularis) during phonological processing and greater engagement of the ventral IFG (pars triangularis) during semantic processing (Brozdowski and Booth, 2021, *preprint*). However, this study focused on single word reading and did not examine connectivity. The current study is the first to directly compare brain-behavior correlations to test how connectivity of the dorsal and ventral pathways may differentially relate to word- and passage- level reading skills.

Meta-analyses of neuroimaging studies across adults and children show high convergence of reading-related activation in the left IFG (Vigneau et al., 2006; Martin et al., 2015). Connectivity between the anterior reading circuit in the IFG and other temporoparietal regions also relate to individual differences in reading skill. For example, the supramarginal and angular gyri in the inferior parietal lobe are thought to be involved in mapping orthographic input to phonological and semantic properties of written words (Welcome and Joanisse, 2012; Lee et al., 2016). Better readers, at the word and sentence level, show greater connectivity between the inferior parietal regions and the left IFG and MTG (e.g., Cutting et al., 2013; Pugh et al., 2013; Aboud et al., 2016; Lee et al., 2016). Yu et al. (2018) observed greater connectivity between left IFG and inferior parietal lobe in 5-year-old children whose phonological abilities increased most over the course of reading development. The strength of these connections predicted later word reading skills at ages 7–8 years old (Yu et al., 2018).

Given that the supramarginal and angular gyri may be involved in both phonological and semantic integration, expanding our posterior mask to include these parietal regions could provide insight into how engagement of the dorsal and ventral pathway differs across tasks (e.g., rhyming versus meaning judgments) in relation to reading skills.

A significant contribution of this study is that we use an individual differences approach to systematically test questions related to the brain bases of reading. First, we used individual functional activation maps to define the seed regions for phonological and semantic processing within each experimental task. We then used individual connectivity maps from the gPPI analyses to examine its relations with children’s reading skills and task performance in the scanner. This approach allowed us to capture variability in the engagement of the reading circuit which is apparent even within populations of skilled readers (e.g., Seghier et al., 2004, 2008; Jobard et al., 2011; Welcome and Joanisse, 2012). In the current sample, variability in the spatial distribution of voxels across the frontotemporal regions of interest is shown in the overlap maps in the Supplementary Figure 1.

In the second exploratory analyses, we used overall task accuracy on the rhyming and meaning tasks instead of standardized reading assessments as the outcome measure of reading skill. The *Word Identification* subtest of the WJ-III primarily assesses children’s oral word decoding but may also engage other processes such as semantics. Similarly, the *Passage Comprehension* subtest involves comprehension skills tapping into vocabulary knowledge, but also involves other processes such as syntax, inferencing, and working memory. We expected accuracy on our experimental word reading tasks might better capture phonological processing, as a core component of word reading skill, and semantic processing, as an essential index of comprehension. Brain-behavior results from these exploratory analyses suggest weak evidence to support the hypothesis that engagement of the dorsal pathway is related to accuracy on the word rhyming task, but no evidence to support the hypothesis that engagement of the ventral pathway is related to accuracy on the word meaning task. Like the previous set of analyses, there was no evidence to support the hypothesis that differential engagement of the dorsal and ventral pathways is related to word rhyming versus word meaning judgments.

Overall, the evidence for our hypotheses is weak and unreliable and therefore needs to be replicated. Thus, we aim to extend these findings to a separate cohort of children and address some of the methodological limitations in the current analyses. Both contrasts we used in the preregistered and exploratory analyses included the fixation condition as the baseline measure. In doing so, the contrasts may have captured general lexical processing effects and/or may not have been sensitive enough to elicit robust engagement of the fronto-temporal regions as related to reading skills. Prior work showing strong associations of brain activation in phonological and semantic hubs with behavioral measures of word- and discourse- level reading skills used non-lexical perceptual stimuli as the baseline subtraction (e.g., Turkeltaub et al., 2003) or contrasted two lexical conditions (e.g., Malins et al., 2016). For example, in participants ages 6–18 years old, phonological awareness ability positively correlated with activation in the left posterior STS (cluster *r* = 0.62) during an implicit reading task that contrasted words with false-font strings (Turkeltaub et al., 2003). Similarly, Malins et al. (2016) tested a “localizer” word reading task to target the orthographic, phonological, and semantic components of reading. Their stimuli type assessing the latter two components were nearly identical to the current study. When contrasting activations pertaining to the semantically related versus unrelated words, engagement of the left IFG (pars triangularis) was related to reading comprehension skills (Malins et al., 2016). In the same study, the authors also observed significant activation in the IFG pars opercularis when contrasting phonologically inconsistent sets of words (O+P–) compared to consistent sets (O+P+), although they did not observe any significant associations between this activation and reading skills. These alternate models may be more sensitive at capturing the phonological and semantic processes that relate to different reading skills.

In conclusion, the present study is the first to directly compare brain-behavior correlations to test how the connectivity of the dorsal and ventral pathways for reading may differentially relate to word- and passage- level reading skills. Our preregistered and exploratory analyses show weak evidence that functional connectivity in the dorsal dIFG-pSTG pathway for phonological processing is positively correlated with word reading skill, but no evidence that connectivity in the ventral vIFG-pMTG pathway during semantic processing is related to reading comprehension skill. Moreover, there was no evidence to support the differentiation between the dorsal pathway’s relation to word reading and the ventral pathway’s relation to reading comprehension skills in children ages 8–15 years old. Our findings need to be replicated with a different sample, and perhaps extended by examining parietal regions implicated in phonological and semantic processing, by using more targeted skill measures of word and passage comprehension and by employing neuroimaging baseline tasks that control more effectively for perceptual processing.

## Data availability statement

Publicly available datasets were analyzed in this study. This data can be found here: https://openneuro.org/datasets/ds002236/versions/1.0.0. Additional materials (e.g., scripts used for data analysis) can be found at https://osf.io/4wdcv/.

## Ethics statement

The studies involving human participants were reviewed and approved by Institutional Review Board at Northwestern University and Evanston Northwestern Healthcare Research Institute. Written informed consent to participate in this study was provided by the participants’ legal guardian/next of kin.

## Author contributions

NW: conceptualization, methodology, formal analysis, and original draft. JB: conceptualization, methodology, review and editing, supervision, and funding acquisition. Both authors contributed to the article and approved the submitted version.

## Footnotes

1
https://osf.io/re7au

2
http://www.fil.ion.ucl.ac.uk/spm

3
http://www.nitrc.org/projects/wfu_pickatlas

4
http://quantpsy.org/corrtest/corrtest2.htm
