# Supplementary material for: Neural pathways of phonological and semantic processing and its relations to children’s reading skills
Source: Front Neurosci. 2022 Oct 12;16:984328. doi: 10.3389/fnins.2022.984328 (PMC9597189; doi:10.3389/fnins.2022.984328)

**Supplementary Table 1**

List of participants included in the current study from the Cross-Sectional Multidomain Lexical Processing dataset available on OpenNeuro.org (Lytle, Bitan, & Booth, 2020).

| sub-01 | sub-36 |
| --- | --- |
| sub-02 | sub-38 |
| sub-04 | sub-41 |
| sub-05 | sub-42 |
| sub-06 | sub-43 |
| sub-07 | sub-44 |
| sub-08 | sub-46 |
| sub-09 | sub-48 |
| sub-10 | sub-57 |
| sub-13 | sub-58 |
| sub-14 | sub-60 |
| sub-16 | sub-61 |
| sub-17 | sub-64 |
| sub-18 | sub-68 |
| sub-19 | sub-71 |
| sub-21 | sub-75 |
| sub-23 | sub-76 |
| sub-24 | sub-77 |
| sub-25 | sub-80 |
| sub-29 | sub-81 |
| sub-31 | sub-82 |
| sub-32 | sub-83 |
| sub-33 | sub-84 |

**Supplementary Figure 1**


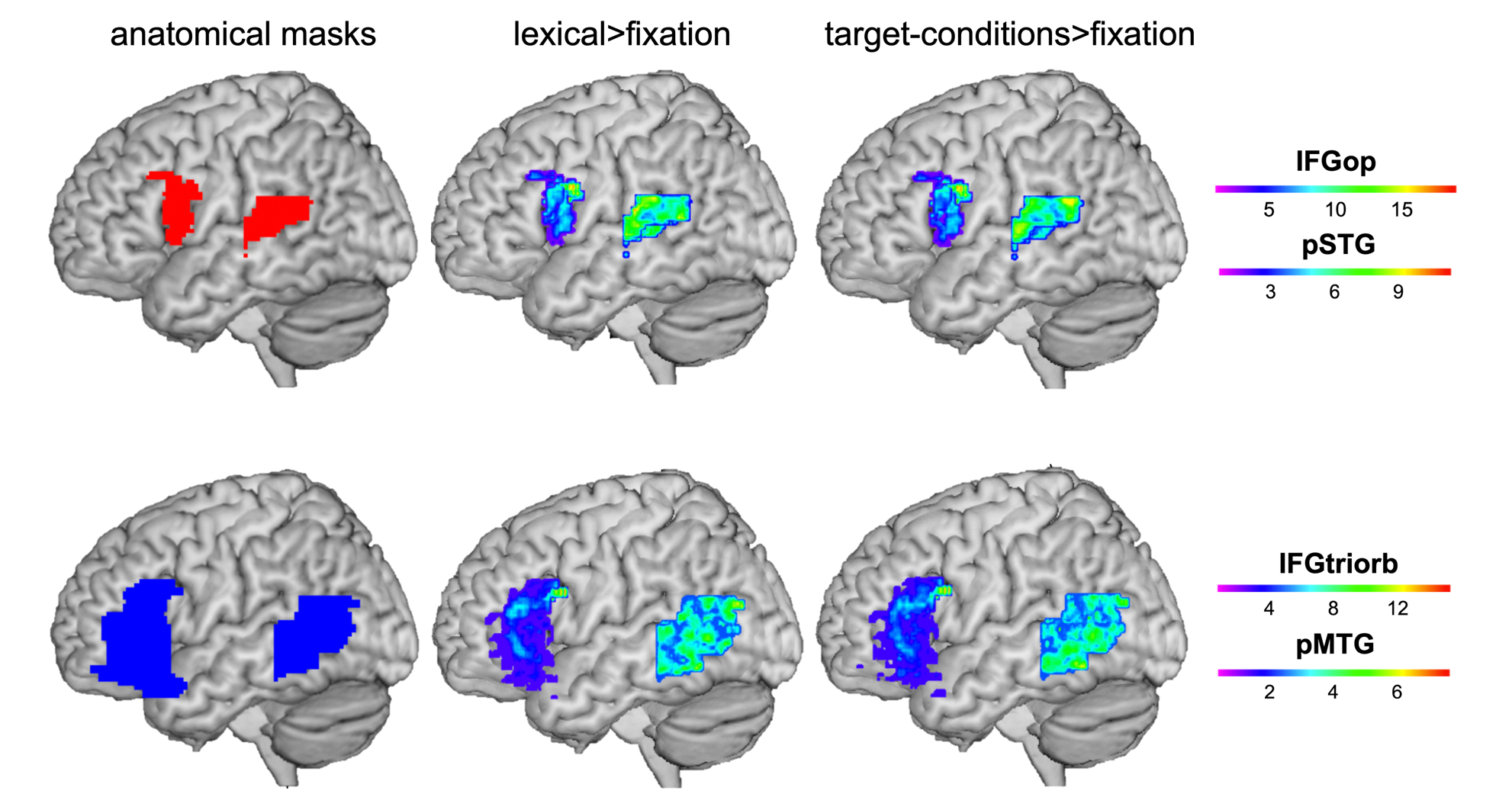
Anatomical masks for the rhyming task (IFG pars opercularis and posterior STG; top row) and the meaning task (IFG triangularis and orbitalis and posterior MTG; bottom row). Spatial overlap across participants within each region-of-interest and analysis type. The color gradient for the frontal regions shows the number of participants with overlapping voxels in first-level analysis (top-activated voxels that make up the seed regions) whereas the color gradient for the temporal regions shows the number of participants with overlapping voxels in the gPPI analysis (the top-connected voxels with its corresponding seed region). Note that the scale indicating overlap is different for each region.

**Supplementary Figure 2**

Whole brain activation maps for the rhyming task for the lexical>fixation contrast with threshold at voxel-wise *p*<0.001 and cluster-wise *p*<0.05 FWE-corrected. Clusters with 5 or more voxels are shown. Cluster-level *k*, peak-level T, and MNI coordinates (x,y,z) are reported in the table below.


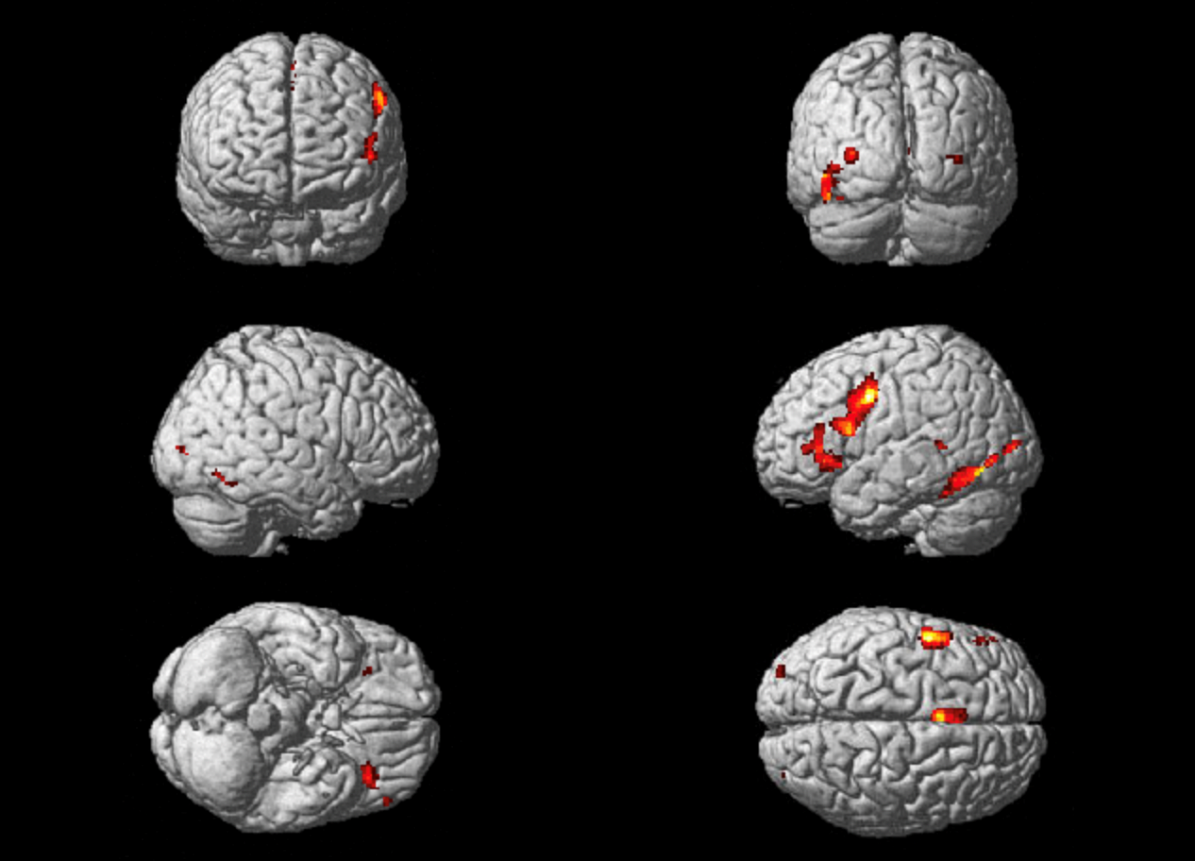


| Region | *k* (2mm) | T | MNI Peak Coordinate | | |
| --- | --- | --- | --- | --- | --- |
| **Left fusiform** | **573** | **11.47** | **-38** | **-48** | **-18** |
| Left fusiform |  | 10.52 | -38 | -56 | -14 |
| Left inferior occipital |  | 9.97 | -44 | -68 | -10 |
| **Left insula** | **427** | **10.96** | **-28** | **24** | **0** |
| Left inferior frontal |  | 7.86 | -44 | 32 | 8 |
| Left inferior frontal |  | 7.76 | -50 | 38 | 6 |
| **Right calcarine** | **173** | **10.73** | **14** | **-74** | **10** |
| **Left precentral** | **632** | **10.01** | **-54** | **0** | **42** |
| Left inferior frontal |  | 9.35 | -46 | 14 | 18 |
| Left precentral |  | 8.91 | -46 | 0 | 34 |
| **Left supplementary motor area** | **313** | **9.83** | **-4** | **16** | **48** |
| Left supplementary motor area |  | 9.80 | -4 | 6 | 60 |
| **Left lingual** | **194** | **8.98** | **-10** | **-74** | **6** |
| **Right fusiform** | **107** | **8.74** | **38** | **-56** | **-16** |
| Right inferior occipital |  | 7.92 | 42 | -64 | -12 |
| **Left middle occipital** | **50** | **8.52** | **-30** | **-88** | **4** |
| **Right middle occipital** | **13** | **8.47** | **36** | **-88** | **4** |
| **Left middle temporal** | **24** | **7.93** | **-46** | **-44** | **8** |
| **Right insula** | **34** | **7.78** | **30** | **28** | **-4** |

**Supplementary Figure 3**

Whole brain activation maps for the rhyming task for the O+P+>O-P- contrast with threshold at voxel-wise *p*<0.001 and cluster-wise *p*<0.05 FWE-corrected. Clusters with 5 or more voxels are shown. Cluster-level *k*, peak-level T, and MNI coordinates (x,y,z) are reported in the table below.


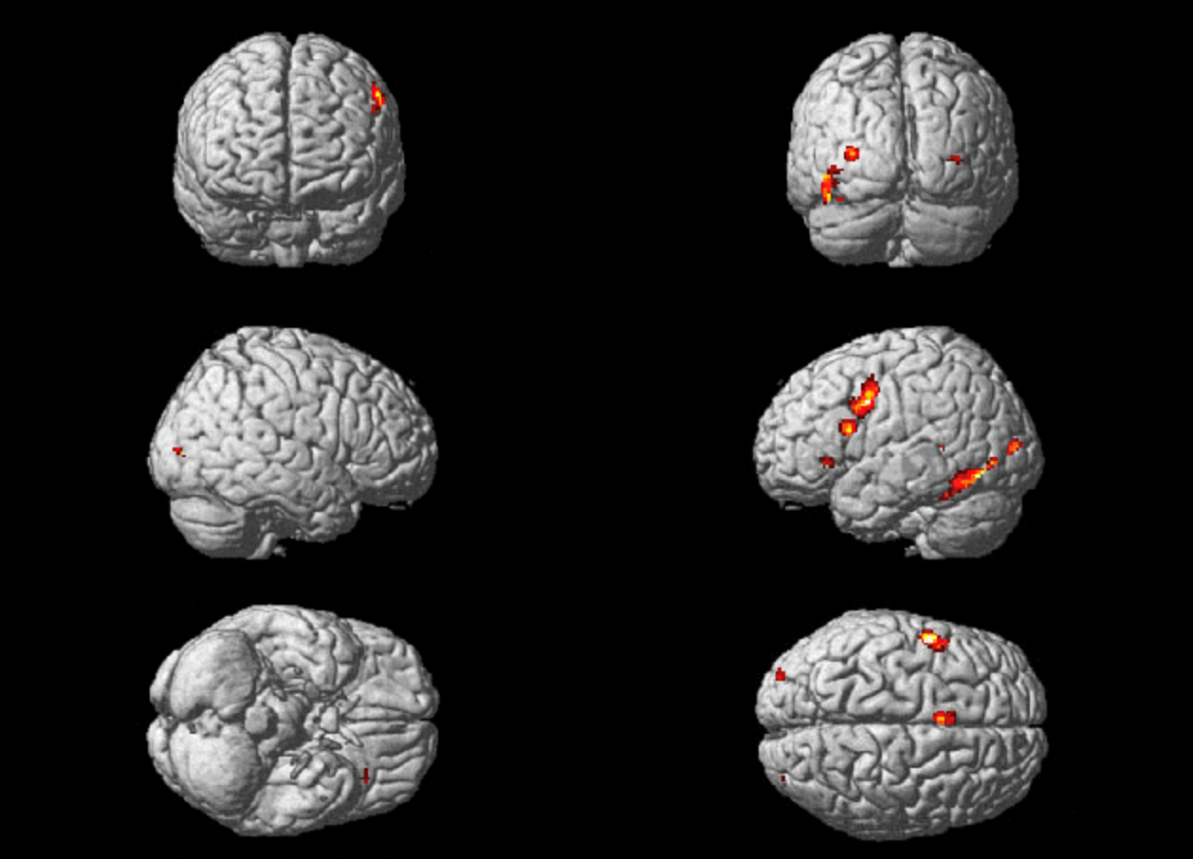


| Region | *k* (2mm) | T | MNI Peak Coordinate | | |
| --- | --- | --- | --- | --- | --- |
| **Left fusiform** | **437** | **10.47** | **-38** | **-48** | **-18** |
| Left inferior occipital |  | 10.03 | -44 | -68 | -10 |
| Left fusiform |  | 9.58 | -44 | -54 | -12 |
| **Left precentral** | **237** | **9.41** | **-56** | **0** | **44** |
| Left precentral |  | 8.27 | -48 | 6 | 32 |
| **Left insula** | **97** | **8.82** | **-28** | **26** | **0** |
| **Left middle occipital** | **49** | **8.64** | **-28** | **-90** | **8** |
| **Left inferior frontal** | **83** | **8.63** | **-46** | **14** | **18** |
| **Left supplementary motor area** | **98** | **8.58** | **-6** | **10** | **52** |
| **Right calcarine** | **72** | **8.56** | **12** | **-72** | **10** |
| **Left lingual** | **75** | **8.42** | **-10** | **-74** | **6** |
| Left calcarine |  | 7.61 | -6 | -82 | 4 |
| **Right middle occipital** | **13** | **8.41** | **36** | **-88** | **4** |
| **Right fusiform** | **26** | **7.51** | **38** | **-56** | **-16** |
| Right fusiform |  | 7.44 | 38 | -48 | -20 |
| **Left middle temporal** | **5** | **7.04** | **-48** | **-44** | **8** |

**Supplementary Figure 4**

| Region | *k* (2mm) | T | MNI Peak Coordinate | | |
| --- | --- | --- | --- | --- | --- |
| **Left insula** | **583** | **12.73** | **-32** | **24** | **0** |
| Left inferior frontal |  | 7.59 | -30 | 28 | -16 |
| Superior temporal pole |  | 7.44 | -50 | 14 | -14 |
| **Left fusiform** | **692** | **12.32** | **-40** | **-62** | **-14** |
| Left fusiform |  | 12.13 | -38 | -52 | -20 |
| **Left supplementary motor area** | **396** | **11.89** | **-2** | **18** | **48** |
| Left supplementary motor area |  | 9.47 | -4 | 6 | 58 |
| Left middle cingulum |  | 7.52 | -6 | 24 | 34 |
| **Left calcarine** | **699** | **11.82** | **-12** | **-72** | **8** |
| Left calcarine |  | 11.67 | -4 | -74 | 10 |
| Right calcarine |  | 10.80 | 8 | -70 | 10 |
| **Left inferior frontal** | **796** | **11.33** | **-42** | **14** | **24** |
| Left inferior frontal |  | 10.03 | -44 | 6 | 28 |
| Left inferior frontal |  | 9.55 | -54 | 16 | 26 |
| **Right fusiform** | **252** | **11.17** | **34** | **-46** | **-20** |
| Right fusiform |  | 10.00 | 40 | -60 | -16 |
| **Left middle temporal** | **303** | **10.31** | **-52** | **-48** | **4** |
| Left middle temporal |  | 9.79 | -60 | -40 | 6 |
| **Right insula** | **144** | **10.30** | **32** | **22** | **-4** |
| **Left middle occipital** | **48** | **8.97** | **-28** | **-88** | **8** |
| **Left thalamus** | **42** | **8.70** | **-10** | **-18** | **12** |
| **Left hippocampus** | **32** | **8.60** | **-22** | **-30** | **-2** |
| **Left superior parietal** | **25** | **8.20** | **-26** | **-56** | **48** |
| **Right inferior occipital** | **18** | **7.99** | **48** | **-78** | **0** |
| **Left middle occipital** | **21** | **7.96** | **34** | **-84** | **4** |
| Right middle occipital |  | 7.85 | 42 | -82 | 2 |
| **Left inferior occipital** | **11** | **7.68** | **-42** | **-76** | **-2** |
| **Left inferior frontal** | **15** | **7.53** | **-54** | **28** | **14** |
| **Left inferior frontal** | **6** | **7.29** | **-54** | **10** | **10** |

Whole brain activation maps for the meaning task for the lexical>fixation contrast with threshold at voxel-wise *p*<0.001 and cluster-wise *p*<0.05 FWE-corrected. Clusters with 5 or more voxels are shown. Cluster-level *k*, peak-level T, and MNI coordinates (x,y,z) are reported in the table below.


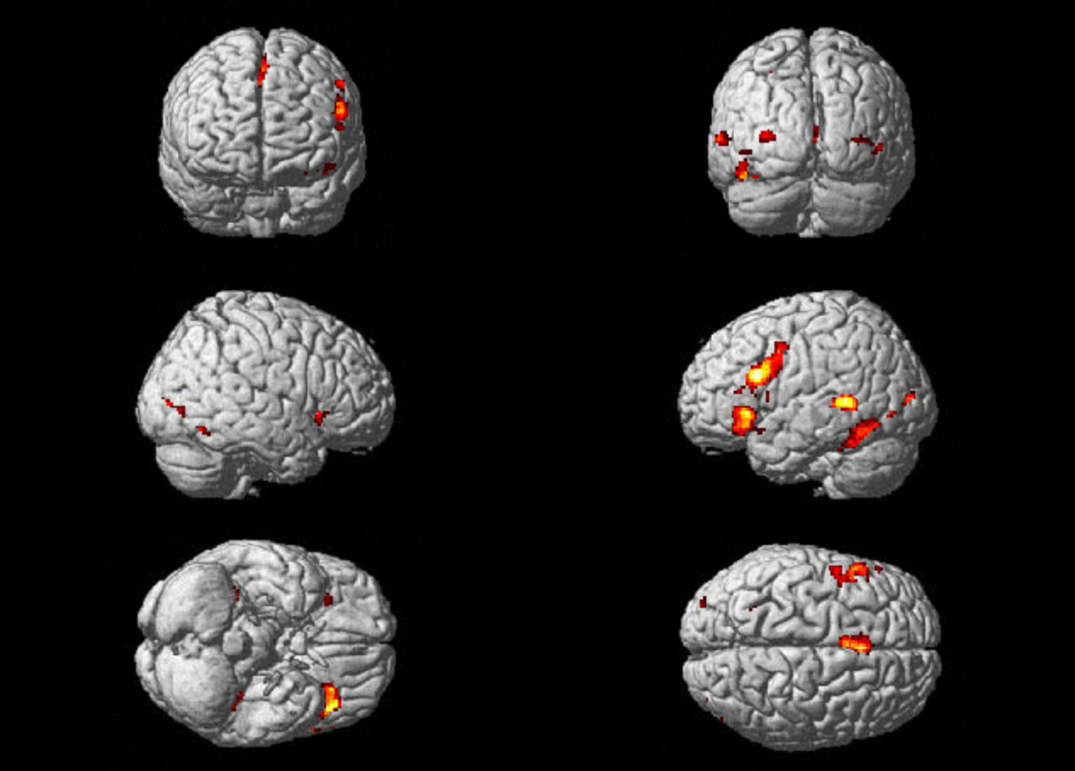


**Supplementary Figure 5**

Whole brain activation maps for the meaning task for the strongly-related>unrelated contrast with threshold at voxel-wise *p*<0.001 and cluster-wise *p*<0.05 FWE-corrected. Clusters with 5 or more voxels are shown. Cluster-level *k*, peak-level T, and MNI coordinates (x,y,z) are reported in the table below.

| Region | *k* (2mm) | T | MNI Peak Coordinate | | |
| --- | --- | --- | --- | --- | --- |
| **Left fusiform** | **620** | **12.73** | **-40** | **-62** | **-14** |
| Left fusiform |  | 11.40 | -38 | -52 | -20 |
| **Left calcarine** | **578** | **11.44** | **-6** | **-74** | **10** |
| Right calcarine |  | 10.63 | 10 | -70 | 10 |
| Left calcarine |  | 7.24 | -12 | -86 | 8 |
| **Left inferior frontal** | **350** | **10.77** | **-38** | **26** | **-4** |
| **Left inferior frontal** | **531** | **10.65** | **-42** | **14** | **24** |
| Left inferior frontal |  | 9.51 | -44 | 6 | 28 |
| Left precentral |  | 8.12 | -54 | 2 | 42 |
| **Left supplementary motor area** | **250** | **10.05** | **-4** | **18** | **46** |
| Left supplementary motor area |  | 9.12 | -4 | 6 | 58 |
| **Right fusiform** | **203** | **9.66** | **36** | **-44** | **-22** |
| Right fusiform |  | 9.39 | 40 | -60 | -14 |
| **Left middle temporal** | **236** | **9.44** | **-52** | **-42** | **4** |
| Left middle temporal |  | 9.20 | -60 | -40 | 6 |
| **Right insula** | **83** | **8.80** | **32** | **24** | **-2** |
| **Left middle occipital** | **38** | **8.46** | **-28** | **-88** | **8** |
| **Left hippocampus** | **7** | **7.86** | **-20** | **-30** | **0** |
| **Left superior parietal** | **17** | **7.84** | **-26** | **-56** | **48** |
| **Right inferior occipital** | **12** | **7.68** | **48** | **-78** | **0** |
| **Left inferior occipital** | **7** | **7.51** | **-42** | **-76** | **-2** |
| **Left thalamus** | **7** | **7.20** | **-12** | **-18** | **8** |


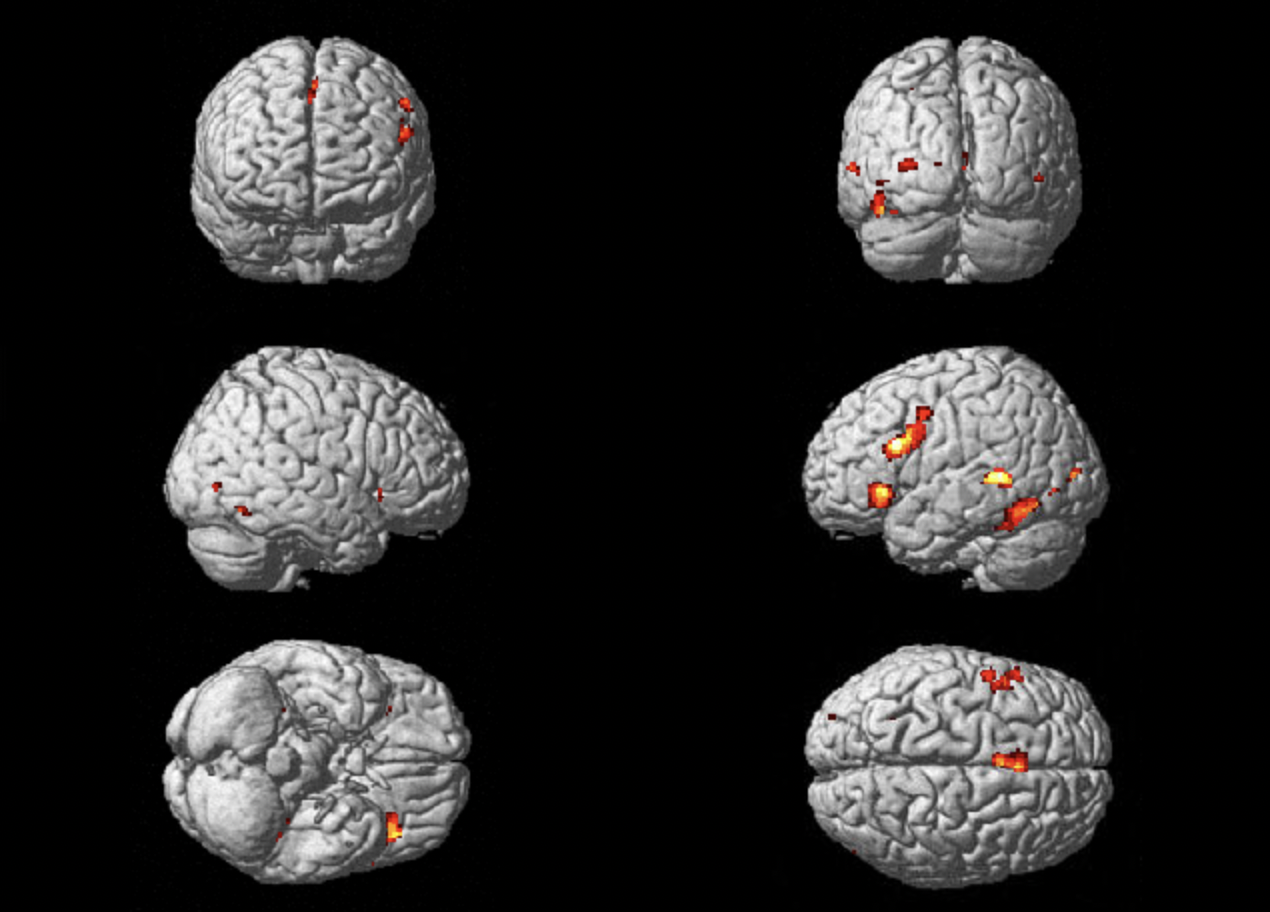

Supplement: Supplementary file 1 [file Data_Sheet_1.docx]
